# Notes of New York Hospital Practice

**Published:** 1874-04-01

**Authors:** 


### NOTES OF NEW YORK HOSPITAL PRACTICE.

*From the New York Medical Record.*

### CHARITY HOSPITAL.

PNEUMONIA. — The remedies commonly employed in this hospital in the treatment of pneumonia, are quinine, carbonate of ammonia, and the alcoholics; occasionally, if the fever is too brisk, liq. ammon. acetatis is administered. The oil-silk jacket is uniformly adopted. Quinine is administered from the beginning. Alcohol, as a rule, is early resorted to. Carbonate of ammonia comes in before the second stage becomes completely developed, and is continued throughout the remaining portion of the course. Diet includes hospital extras. An effort was made by one of the visiting physicians to withdraw, somewhat at least, from this highly tonic and stimulating plan of treatment. Accordingly, liq. ammon. acetatis and tincture of aconite were recommended as the chief remedies to be employed during the earlier part of the disease; but the experiment proved so disastrous, the rate of mortality increasing so rapidly, that the attempt at reformation was at once abandoned.

The constitutional condition of the patients who find admission to this hospital, doubtless has a controlling influence upon the treatment necessary to be adopted in this class of diseases, if the best results would be obtained.

EXPECTORANT MIXTURE.—An expectorant mixture very commonly used in cases of chronic bronchitis, and with very good results, is the following: Ammon. muriat.; liq. morph. sulph. (Mag.), of each one drachm; syr. tolu; syr. scillæ co., of each one ounce. Mix. S. one drachm, t.i.d.

NIGHT-SWEATS OF PHTHISIS.—House-Physician Smith remarked that two grain pills of oxide of zinc t.i.d., has answered a better purpose in his division for controlling this symptom than any remedy that had been employed.

ACUTE ARTICULAR RHEUMATISM.—Dr. Smith also directed my attention to an external application to be used for the joints, during the progress of this affection. The following is the formula: Tinct. opii, one ounce; spts. chloroform, one and a half ounce; lin. saponis, ad., one pint. Mix. This liniment is applied freely over the joints, and immediately covered with cotton and oil-silk. The relief from pain afforded by this application has been very gratifying to all his rheumatic patients. The general treatment is alkaline.

IRRITABLE STOMACHS.—The case to which my attention was directed, was one in which the ordinary irritability of stomach associated with phthisis, required special treatment. The method of treatment, however, is almost uniformly adopted when an irritable condition of the stomach manifests itself in connection with *any* chronic disease. The remedy is *raw beef*, chopped fine, and seasoned with salt, pepper, and vinegar. The patient is to subsist entirely upon beef prepared in this manner. Dr. Smith remarked that this plan had, in his wards, seldom failed to afford relief to this condition, when associated with any chronic affection.

SILICATE OF SODA IN THE TREATMENT OF FRACTURES.—House-Surgeon Pierce informed me that he had employed the silicate of soda in his division in the treatment of fractures equally as much as he had employed PLASTER-OF-PARIS. The soda splint has furnished very pleasing results, and, when carefully applied, makes a most elegant and serviceable splint. Three bandages are ordinarily used, the limb being coated over with the silicate, after the application of each bandage. It is also well, and perhaps always advisable, to add narrow strips of pasteboard as the bandages are being applied. Extension, in the proper direction, must be maintained until the splint is thoroughly dried.

ACETIC SPRAY IN DIPHTHERIA.—Diphtheria, scarlet fever, typhus and typhoid fevers, and small-pox, constitute a group by themselves.

By present arrangement this department falls under charge of the hospital staff, as one of the branches of "Out-door Service." Dr. Partridge, House-Physician, mentioned that, with regard to diphtheria, very satisfactory results had been obtained in the local treatment of the disease by the use of acetic acid, in solutions of varying strength, in the form of spray. The remedy is used by means of the so-called atomizer. It seems to have the power to dissolve the membrane, and in several cases, where well-developed and somewhat advanced croupy symptoms were present, all were relieved, and that quite speedily, by the use of this agent. The administration of alcoholics is governed by the condition of pulse and temperature. The rate of mortality is small.

ITCHING AND PITTING IN SMALL-POX.—To relieve the intense itching which attends this eruption, washing the surface with glycerine and water acts as if by magic.

To prevent pitting, one of the visiting physicians recommended the use of tr. iodine. The remedy should be employed, if possible, before vesicles are formed. It is to be applied once a day. The effect of this remedy has not been sufficiently noted in the Small-Pox Hospital to warrant any conclusion relative to its value in this direction.

It was used, in one case, after the eruption had been vesicular for one or two days, but before it had become pustular; and only a moderate amount of pitting followed. Whether the adoption of an *ectrotic* plan of treatment will not do the patient more harm than can be counterbalanced by the benefit arising from a moderate arrest of pitting, or even a complete prevention of pitting, is, in many cases, thought to be a question worthy of consideration.

To prevent the formation of abscesses, the combined hypophosphites have served a very excellent purpose. One patient had eight abscesses, and another four, at various situations on the body, and all had rapidly healed under the influence of this combination treatment. In several instances, threatened formation of abscess had been dispelled. The influence of this remedy, therefore, was looked upon with favor, for the reason that abscesses, under such circumstances, are not infrequently attended with *grave* results.

### BELLEVUE HOSPITAL.

HÆMORRHAGES.—This class of difficulties, such as hæmoptysis, hæmorrhage from bowels, hæmaturia, hæmatemesis, and hæmorrhages from the uterus, are treated upon this division most satisfactorily by the use of spirits of turpentine. The remedy is commonly administered in ten drop doses, repeated every two hours.

IODIDE OF POTASSIUM IN ANEURISM.—Our attention was directed to a case of thoracic aneurism, in which nearly all the distressing symptoms had disappeared since the patient had been placed upon large doses of iodide of potassium.

PNEUMONIA.—Quinia will reduce the temperature and pulse of pneumonia, with a good deal of certainty. Such an effect is beneficial. It requires large doses to accomplish this. It is customary to give pneumonic patients ten grains in the morning, and fifteen grains at night, or ten grains morning and night.

CARBOLIC-ACID OINTMENT FOR SCABIES. — Its success warrants farther trial.

MONARTHRITIC CASE. — Cardiac complication had occurred since admission to the hospital. The case shows the importance of correct diagnosis, in order that the patient may receive the greatest benefit from treatment.

HÆMORRHAGE FROM THE LUNGS. —There were some features of interest in connection with this case. The patient was aged twenty-eight, single, and a domestic. Mother died of consumption. One year previous to her admission, suffered from a sudden attack of hæmorrhage from the lungs. Soon after she began to lose strength, had night-sweats, and lost her appetite. Pain, especially beneath the scapula. Has suffered from several severe hæmorrhages. She has mitral regurgitation.

Hæmoptysis with mitral regurgitation, is a point of interest.

Mitral obstruction is the usual cause of hæmoptysis, when dependent upon cardiac disease.

If the hæmorrhages are profuse, it is hardly warrantable to conclude that they are due to cardiac lesion.

If regurgitant lesion alone exists, it is never warrantable to say that the hæmoptysis is dependent upon a cardiac cause.

The patient has improved since her admission to the hospital.

IMPROVEMENT IN A CASE WHERE THERE HAS BEEN REPEATED AND PROFUSE HÆMORRHAGES FROM THE LUNGS.—This is another point of interest. In general, patients do better when hæmorrhages from the lungs occur, if they are phthisical patients; and they are also rather more likely to recover than those who do not have such hæmorrhages; or, if they do not recover, they are apt to have an arrest of the disease. These facts can be used for the encouragement of patients.

Such were some of the interesting features of the case we were visiting, as drawn out by the visiting physician.

